# Melanonychia: A Challenging Green Nail Syndrome Case Report

**DOI:** 10.1002/ccr3.70723

**Published:** 2025-08-18

**Authors:** Kyoko Sugioka, Tomoko Akeda, Keiichi Yamanaka

**Affiliations:** ^1^ Department of Dermatology Mie University Graduate School of Medicine Tsu Mie Japan

**Keywords:** bacterial culture, green nail, nadifloxacin, *pseudomonas aeruginosa*

## Abstract

Nail dyschromia caused by *Pseudomonas aeruginosa* infection, known as Green Nail Syndrome, is a condition occasionally encountered by clinicians. It usually affects one or two nails, but can involve more. Dyschromia may be limited to the nail tip. Treatment with topical nadifloxacin proved to be effective.

A 19‐year‐old woman presented with a 6‐month history of slowly progressive dyschromia of both toenails (Figure 1A,B). Similar symptoms extended to the nails on her hands. The dyschromia was dark brown with a greenish hue. The patient had no significant medical history, no exposure to acrylic nails, and no history of soil work. Initial treatments at previous physicians with oral antibiotics and topical steroids were ineffective. Laboratory evaluations, including fungal examination, occult blood testing, and bacterial culture, were negative. Based on clinical suspicion of green nail syndrome, often caused by *Pseudomonas aeruginosa* infection, treatment with 1% topical nadifloxacin was initiated. After one month, the lesions resolved (Figure 1C).

**FIGURE 1:**
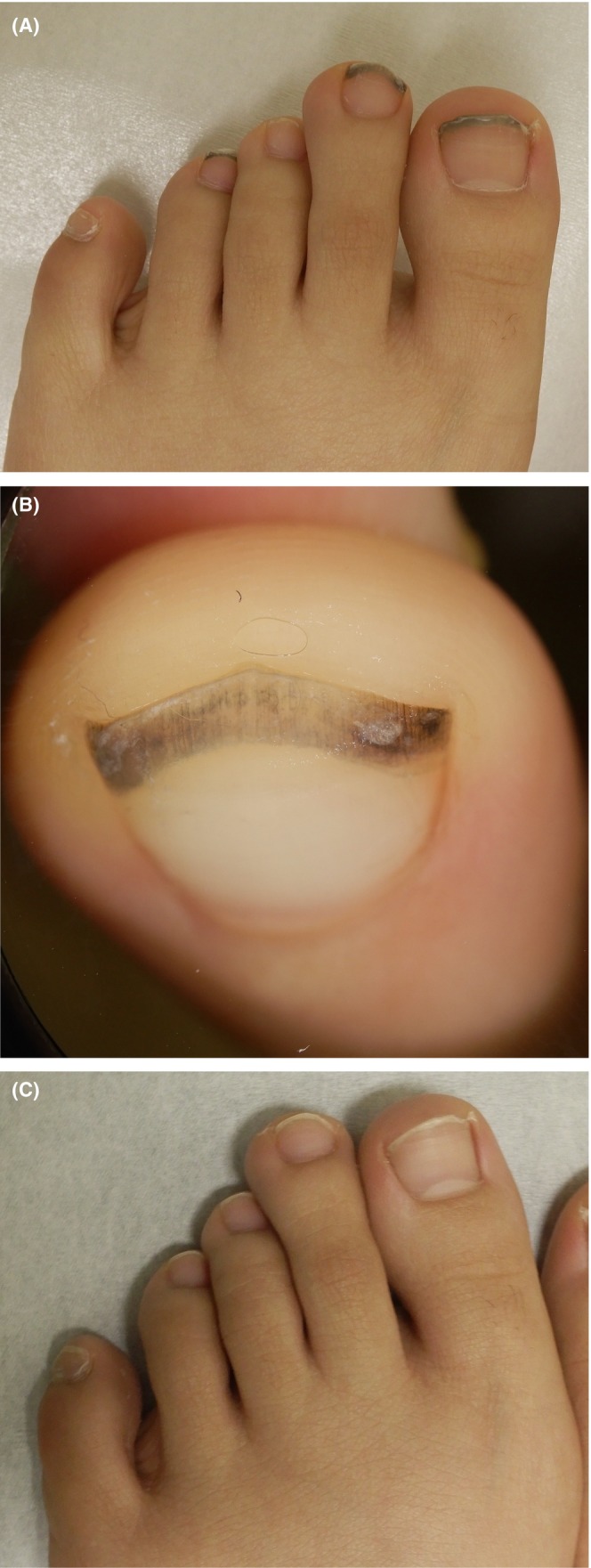
(A) Initial presentation showing dark brown to greenish dyschromia at the distal nail plates. (B) Dermatoscopic examination revealed a black dyschromia localized to the free edge of the nails. (C) Resolution of dyschromia n one month after initiation of 1% topical nadifloxacin.

Green nail syndrome is a persistent greenish pigmentation of the nail plate mainly due to *Pseudomonas aeruginosa* infection, originally described in 1944 by Goldman and Fox. The differential diagnosis of green nail includes subungual hematoma, malignant melanoma, and exogenous yellow pigment exposure. The patients with green nail often have a history of compromised immune function, frequent exposure to water, soap, or detergents, and prior nail problems such as onycholysis, paronychia, psoriasis, or trauma. Bacterial cultures for green nail often yield false negatives [1], as is in this case. Although green nail syndrome typically affects one or two nails [2], the current case suggests trauma may have predisposed nails to infection and spread to other nails, leading to a widespread presentation. This case highlights the importance of considering *Pseudomonas aeruginosa* infection in the differential diagnosis of nail discoloration, despite negative initial tests, and demonstrates rapid, effective, complete resolution with therapy.

## Author Contributions

**Kyoko Sugioka:** conceptualization, investigation, methodology, writing – original draft. **Tomoko Akeda:** conceptualization, writing – original draft. **Keiichi Yamanaka:** conceptualization, investigation, writing – review and editing.

## Ethics Statement

The research was conducted in accordance with the Declaration of Helsinki. The paper is exempt from ethical committee approval because of the single case study.

## Consent

The patient gave us consent for his photographs and medical information to be published in print and online with the understanding that this information is publicly available. Written consent for publication was obtained from the patient.

## Conflicts of Interest

The authors declare no conflicts of interest.

## Data Availability

The patients data are not publicly available on legal or ethical grounds.

## References

[1] T. Cosio , R. Petruccelli , R. Gaziano , et al., “Green Nail Syndrome Treated With Ozenoxacin: Two Case Reports,” Case Reports in Dermatology 15, no. 1 (2023): 217–224. 38023344 10.1159/000533923 PMC10653707

[2] S. Geizhals and S. R. Lipner , “Retrospective Case Series on Risk Factors, Diagnosis and Treatment of *Pseudomonas aeruginosa* Nail Infections,” American Journal of Clinical Dermatology 21, no. 2 (2020): 297–302. 31595433 10.1007/s40257-019-00476-0

